# Structure-Function of the High Affinity Substrate Binding Site (S1) of Human Norepinephrine Transporter

**DOI:** 10.3389/fphar.2020.00217

**Published:** 2020-03-05

**Authors:** Prerna Jha, Lotten Ragnarsson, Richard J. Lewis

**Affiliations:** Institute for Molecular Bioscience, The University of Queensland, Brisbane, QLD, Australia

**Keywords:** monoamine transporter, docking guided mutagenesis, noradrenaline uptake, molecular modeling, structural determinants

## Abstract

The human norepinephrine transporter (hNET) is a member of the neurotransmitter/sodium symporter family, which also includes the neuronal monoamine transporters for serotonin (SERT) and dopamine (DAT). Its involvement in chronic pain and many neurological disorders underlies its pharmaceutical importance. Using the X-ray crystal structures of the human serotonin transporter (hSERT) (PDB 5I6X) and *Drosophila melanogaster* dopamine transporter (dDAT) (PDB 4M48 and PDB 4XPA) as templates, we developed molecular models for norepinephrine (NE) bound to its high affinity binding site (S1) in the hNET. Our model suggests that the S1 site for NE is deeply buried between transmembrane helices (TMHs) 1, 3, 6, and 8 and overlaps the binding site for leucine in the bacterial leucine transporter (LeuT) and dopamine (DA) in dDAT. Mutational studies identified the functional binding pocket for NE comprised residues A73, A77, N78, V148, N153, I156, G320, F329, N350, S420, G423, and M424, which all influenced NE affinity and/or transport. These effects support a NE-hNET docking model where A73, A77, G320, S420, G423, and M424 form H-bond interactions with NE, V148, I156, and F329 form hydrophobic interactions with NE, whereas N78 affects NE transport and N350 affects NE affinity and transport via an influence on the octahedral co-ordination of the Na_1_^+^ ion. Consistent with a conserved structure-function amongst sodium-dependent neurotransmitter transporters, S1 residues A73, A77 (G100 in hSERT), N78, V148 (I150 in hSERT), N153, G320, F329 (Y331 in d DAT), N350, and G423 are conserved in DAT and SERT, indicating they likely play conserved functional roles.

## Introduction

Spatio-temporal regulation of NE neurotransmission, presynaptic homeostasis and maintenance of extra synaptic monoamine levels is maintained by the NET (Torres et al., 2003). The NET belongs to the solute carrier (SLC) 6 gene family, also known as the neurotransmitter sodium symporters (NSSs), which facilitate sodium- and chloride-dependent transport of NE into pre-synaptic neurons (Masson et al., 1999; Chen et al., 2004). The SLC6 gene family includes the monoamine [DA, NE, and 5-HT], amino acid [GABA, glycine, and proline] and osmolyte (betaine, creatine, and taurine) transporters (Dougherty et al., 1999; Jayanthi and Ramamoorthy, 2005; Klimek et al., 1997). The human NET (hNET) is of particular clinical relevance because dysregulated NE neurotransmission plays a key role in diseases such as chronic pain (Torebjörk et al., 1995), depression (Klimek et al., 1997), OCD (Hollander et al., 1988), anxiety, ADHD (Dougherty et al., 1999), cardiac diseases (Jayanthi and Ramamoorthy, 2005), obesity, and orthostatic intolerance (Hahn et al., 2003; Zhou, 2004). Several studies have identified hNET single nucleotide polymorphisms (SNPs) and their associated pathologies. These studies shed light on how alterations in the protein’s structure, expression and/or function lead to disease (Supplementary Table S1). Thus, alterations in the structure, function and expression of this transporter can produce significant anatomical and functional sequelae.

In 2005, the first high resolution transporter structure was solved for the bacterial (*Aquifex aeolicus*) LeuT (Yamashita et al., 2005). This structure shows the substrate leucine and the two Na^+^ ions bound to the interior core of the transporter, now known as the primary or S1 of the transporter. The LeuT crystal structure (PDB ID 2A65) also helped identify a second low affinity binding site (S2) of NE at hNET (Wang et al., 2012). More recently, the crystal structure of the *Drosophila melanogaster* dopamine transporter (dDAT) bound to the tricyclic antidepressant nortriptyline was solved at 2.95 Å (Penmatsa et al., 2013) and dDAT bound to its substrate DA was solved at 2.95 Å (Wang et al., 2015). Interestingly, the dDAT structure is in an outward-open conformation with DA bound to S1 where it is surrounded by TMHs 1, 3, 6, and 8 (Wang et al., 2015). The amine group of DA interacts with the carboxylate of D46 at a distance of 3 Å, whereas the catechol group of DA binds into a cavity formed by residues A117, V120, D121, Y124, S422, and F325. The catechol ring of DA interacts with TMHs 3 and 8 by forming hydrogen bonds with the carboxylate group of D121 (Wang et al., 2015). In 2016, crystal structures at 3.15 Å of the outward open hSERT bound to paroxetine or (S)-citalopram using a transport-deficient variant of hSERT (Coleman et al., 2016) revealed the allosteric binding site for antidepressants. However, high resolution structures of hNET and hDAT remain to be elucidated, and understanding the structure-function of these transporters still requires homology models to guide interpretation at the molecular level.

Identification of functionally significant ligand interactions have mostly focused on the inhibitor site, with residues affecting cocaine (Kitayama et al., 1992) and its analogs benztropine and GBR12909 binding to DAT (Beuming et al., 2008; Schmitt et al., 2008) and antidepressants [(S)-citalopram] and cocaine binding to SERT (Chen et al., 1997; Andersen et al., 2010; Sarker et al., 2010; Sørensen et al., 2012) identified. Whereas a number of binding site residues for DA in DAT (Kitayama et al., 1992; Beuming et al., 2008), 5-HT in SERT (Chen et al., 1997; Field et al., 2010) and NE in NET (Schlessinger et al., 2011; Koldsø et al., 2012) have also been identified, a comprehensive study of all residues potentially contributing to substrate (NE) binding and transport at NET has not been undertaken. In this study, homology-models of hNET were constructed based on the crystal structures of dDAT (PDB ID 4XPA and 4M48) and the hSERT (PDB ID 5I6X) as templates to identify the possible NE binding site residues in the high affinity binding pocket. To identify the high affinity binding site of NE in hNET (S1), NE was docked to hNET using Molegro Virtual Docker (MVD) software package (Molegro ApS, Aarhus, Denmark). Docking-guided site directed mutagenesis combined with functional and binding assays revealed that residues A73, A77, N78, V148, N153, I156, G320, F329, N350, S420, G423, and M424 contributed to NE affinity, transport and/or surface expression. Understanding NE-hNET interactions at a molecular level revealed a number of functionally conserved residues across the SLC 6 family (Supplementary Table S2) that might facilitate the design of transporter inhibitors with improved selectivity.

## Materials and Methods

### Materials

Dulbecco’s Modified Eagle’s medium (DMEM) and fetal bovine serum were purchased from Invitrogen (Carlsbad, CA, United States). Cell dissociation buffer was purchased from Life Technologies. Nisoxetine (NX), mutation and sequencing primers were purchased from Sigma-Aldrich. Cell culture dishes and 96-well plates were purchased from Nunc (Roskilde, Denmark). [^3^H]-NE (40–80 Ci/mmol), [^3^H]-NX (70–87 Ci/mmol), OptiPhase Supermix cocktail and Betaplate Scintillant were purchased from PerkinElmer Life Sciences (Waltham, MA, United States).

### Structural Alignment Between hNET, hSERT, dDAT, and LeuT

**FIGURE 1:**
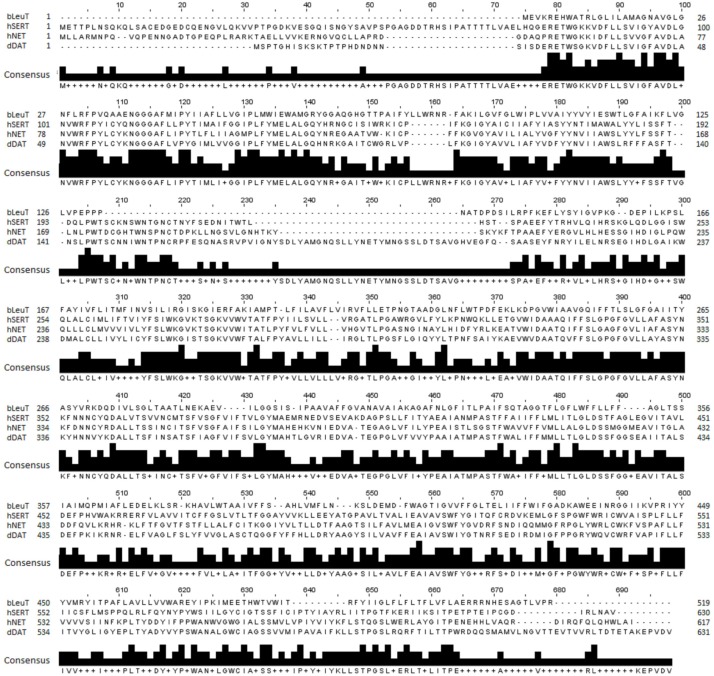
Sequence alignment of hNET, LeuT (PDB ID 2A65), dDAT (PDB ID 4M48), and hSERT (PDB ID 5I6X) used for hNET model creation.

An optimal sequence alignment between hNET and hSERT, dDAT and LeuT is pivotal for generating an accurate model of the hNET. hNET shares a low sequence identity of 24% with the bacterial homolog LeuT, a sequence identity of 56% with dDAT and 53% with hSERT. A multiple sequence alignment for hNET, LeuT, hSERT, and dDAT was generated using ClustalW as shown in Figure 1 (Larkin et al., 2007). The alignment was then manually adjusted based on the recently published alignments (Haddad et al., 2016).

### hNET Model Generation

A total of 15 hNET models were constructed, with five based on the outward dDAT crystal structure (PDB ID 4M48), five based on the partially occluded dDAT crystal structure (PDB ID 4XPA), and five models based on the hSERT crystal structure (PDB ID 5I6X). MODELLER 9.11 was used to build the hNET homology models (Fiser and Šali, 2003).

### Physics-Based Evaluation

MolProbity (Chen et al., 2010) was used to evaluate Ramachandran outliers, rotamer outliers, Cβ deviations (>0.25 Å), bond lengths, bond angles, chiral volumes, planar groups, and clashes.

### Knowledge-Based Evaluation

Qualitative model energy analysis (QMEAN) (Benkert et al., 2008) was used to compare three general statistical potential terms covering the stability of protein structure in addition to secondary structure and solvent accessibility parameters.

### hNET-NE Docking

Molegro Virtual Docker (MVD) (Molegro, 2011) was used to dock NE into the S1 site of hNET given its reported high accuracy and versatility (Chen et al., 2011). The bond order and the atom types of both the NE and the hNET were automatically corrected and assigned the appropriate charges during their preparation. A cavity detection algorithm was used to detect the potential binding pockets and MolDock [GRID] (Molegro ApS, Aarhus, Denmark) used as the scoring function with a grid resolution of 0.30 Å. The MolDock SE algorithm was applied for 10 runs with a maximum population size of 50 and 1500 iterations. The energy threshold was set at 100 and the simplex evolution of maximum steps and neighbor distance factor set at 300 and 1.00, respectively. The best conformations were selected based on the lowest docked energy, and the LPC/CSU server used to analyze the ligand-protein contacts (Sobolev et al., 2005).

### Site-Directed Mutagenesis

Generation of point mutations in the hNET cDNA in the mammalian expression plasmid pEUK-C1-amp (pEUK-C1-amp-hNET) was performed by site-directed mutagenesis using the QuikChange mutagenesis kit (Stratagene Cloning Systems, La Jolla, CA, United States) according to the manufacturer’s protocol. A series of mutation primers were designed to produce point mutations of the predicted S1 site residues in hNET (F72A, A73G, A73L, A73R, A73V, A77L, A77R, N78A, V148A, N153A, I156A, S318A, G320A, F323A, V325A, F329A, N350A, S420A, G423A, and M424A). All non-alanine residues were conservatively mutated to alanine, while conservative A73G and A73V hNET mutants were made to understand the function of A73. In addition, A73L, A73R, A77L, and A77R mutants were also constructed to study the influence of size and polarity at these positions. Plasmids were purified from overnight TOP-10 *E. coli* cultures (Invitrogen) grown in LB media supplemented with 100 mg/ml ampicillin using the PureLink Quick Plasmid Miniprep Kit (Invitrogen). Mutated DNA was sequenced by the Australian Genome Research Facility to confirm the correct sequence was obtained for each mutation.

### Cell Culturing and Transient Expression of hNET

COS-7 cells (American Type Culture Collection, Manassas, VA, United States) were cultured in growth medium (DMEM supplemented with 10% v/v fetal bovine serum) at 37°C in a humidified 5% CO_2_ environment. Cells were transiently transfected with purified plasmid DNA encoding WT or mutant hNET using FuGENE HD (Roche) following the manufacturer’s protocol. Briefly, 15 μg of DNA and 75 μl of transfection reagent were mixed in 0.5 ml of DMEM and incubated at room temperature for 20 min before addition to a T-75 culture flask containing confluent COS-7 cells.

### Membrane Preparation

Membrane preparations were conducted 48 h post transfection. COS-7 cells were scraped from T-75 flasks and washed three times with hNET assay buffer by repeated suspension of the cells followed by centrifugation at 467 × *g* to remove the media. The washed cells were finally resuspended in hNET assay buffer containing 1.6 mg/ml of protease inhibitor cocktail (Roche Diagnostics, United States) and lysed by sonication. Cell debris, including the nuclei, was removed by centrifugation at 467 × *g* for 10 min. The supernatant, containing the hNET-expressing cell membranes, was centrifuged again at 39000 × *g* for 35 min at 4°C. The cell membrane-containing pellet was resuspended in hNET assay buffer, protein concentration determined using a Bradford assay kit (BioRad), and used within 24 h.

### SDS-Polyacrylamide Gel Electrophoresis (SDS-PAGE) and Western Blot

SDS-polyacrylamide gel electrophoresis was performed to confirm the expression of the WT or mutant hNET protein. The SDS polyacrylamide gels were made with an upper 3% stacking gel and a lower 10% running gel. Samples to be analyzed were prepared using 20 μg of cell membrane protein with 5 μl loading dye (0.225 M Tris–HCl, pH 6.8; 50% (v/v) glycerol, 5% (w/v) SDS, 0.05% (w/v) bromophenol blue; 500 mM β-mercaptoethanol). Samples were incubated for 20 min at 37°C before loading on the gel along with the protein molecular weight standards. Electrophoresis was carried out at a constant voltage of 180 V for 40 min until the dye front reached the bottom of the gel. The gel was then removed from the electrophoresis apparatus. The gels were soaked in electroblot buffer (14.4 g glycine, 3.03 g Tris base, 10% methanol) for 1 min and then electroblotted onto a nitrocellulose membrane (BioRad). The gels were then placed on the membrane and sandwiched between two layers of filter paper and sponges in a cassette. The cassette and the ice blotting unit were placed in the electroblotting apparatus and filled completely with electroblot buffer. Transfer was conducted at 60 V for 90 min and the membranes were then placed in blocking solution [5% (w/v) skim milk] for 1 h. The primary sequence-specific antibody (mouse monoclonal antibody to NET targeting N-terminal amino residues 17–33, Abcam, United States) was added in 1:3000 dilution in blocking solution and incubated for 45 min on a shaker. Excess antibody was then removed by washing three times in blocking solution for 5 min each. The secondary antibody [fluorescently labeled goat anti-mouse IgG (H + L) Alexa Fluor 680, Molecular Probes] was then added in 1:2500 enzyme to blocking solution and allowed to bind for 45 min. The excess secondary antibody was removed with three 1 min washes with PBS. The membrane was scanned using Odyssey IR imaging system (LI-COR Biosciences). As is the case for most membrane proteins, they generally migrate faster in SDS-PAGE as an artifact of SDS-lipid micelle complex. The molecular weight of NET WT in total expression is observed as ∼54 kDa in SDS-PAGE, consistent with literature reports (Hahn et al., 2003).

### [^3^H]-NX Binding Measurements

Saturation binding experiments were performed to determine the B_max_ and K_d_ for NX at each of the mutants. Reactions containing membranes from hNET-transfected COS-7 cells (20 μg protein) and increasing concentrations of tritiated NX [(^3^H]-NX)] (0.125–50 nM) in assay buffer were established in clear round bottom 96-well plates. Each assay was performed in triplicate in a total reaction volume of 150 μl. The non-specific binding of [^3^H]-NX was determined in the presence of 200 μM NX. After 1 h incubation on ice, the membranes were harvested onto Whatman GF/B filtermats (PerkinElmer) pre-treated with 0.6% polyethylenimine using a Tomtec harvester. BetaPlate scintillant (PerkinElmer) was then applied and the filter-bound radioactivity detected using a Wallac MicroBeta (PerkinElmer). Specific [^3^H]-NX binding was calculated as the difference between the total and the non-specific binding. Each experiment was performed in triplicate and repeated 3–4 times. Total measured counts were always<10% of the counts added in all binding experiments.

### [^3^H]-NE Transport Measurements

Adherent transfected cells were washed with 10 ml of phosphate-buffered saline (PBS) 24 h post transfection. The cells were then detached with 2 ml of Cell Dissociation Buffer Enzyme-Free PBS-based (Life technologies) followed by suspension of cells in growth medium. The cells were then added to 96-well plates at a density of 10,000 cells/well. Uptake assays were performed 24 h post plating to determine V_max_ and the apparent Michaelis constant K_m_, defined as the extracellular NE concentration required for half-maximal transport velocity (V_max_). Cells were washed manually with 100 μl/well of hNET assay buffer (25 mM HEPES, 125 mM NaCl, 1.2 mM MgS0_4_, 4.8 mM KCl, 1.2 mM KH_2_PO_4_, 1.3 mM CaCl_2_, 5.55 mM D-(+)-glucose, 1 mM ascorbic acid, pH 7.4). Uptake was initiated by the addition of 50 μl/well of hNET assay buffer containing increasing concentrations of [^3^H]-NE (40.5 Ci/mmol, PerkinElmer) from 31 nM–5 μM in triplicate. Non-specific uptake was measured using 200 μM of unlabeled NX. The uptake was abolished after 10 min incubation at 37°C by washing the cells twice with 100 μl/well of hNET assay buffer. Complete cell lysis was obtained by adding 50 μl of 0.1 M NaOH/well followed by 1 h incubation. Accumulated radioactive neurotransmitter was quantified using a Wallac MicroBeta counter (PerkinElmer Life Sciences). In each of the uptake experiment, the total measured counts were always <10% of the counts added.

### Statistics and Data Analysis

Curve fitting of saturation binding and transport kinetic data was performed by non-linear regression using the software package Prism (GraphPad Software). The B_max_ and K_d_ values were determined from saturation bindings curves, whereas V_max_ and K_m_ values were determined from saturation uptake curves. Data are presented as means ± SEM of results obtained from 3–15 separate experiments, each performed in triplicates. For multiple comparisons, one-way analysis of variance (ANOVA) was used with *post hoc t*-tests performed by Dunnett’s method using Prism (GraphPad Software). Values of *P* < 0.05 were considered significant. The calculations and statistical analysis on the K_d_ and K_m_ data were performed on the log values.

## Results

### Evaluation of hNET Models

Evaluation of physical properties of homology models focuses on the problems resulting from protein structure outliers and steric clashes. Molprobity allows an all-atom contact analysis and quality check for identification of physical anomalies (see Table 1), with the list of backbone outliers for hNET summarized in Table 2. Knowledge-based evaluation by qualitative model energy analysis (QMEAN) (Benkert et al., 2008) additionally revealed deviations in all-atom pairwise energy, solvation energy and torsion angle energy. Based on these evaluations, we selected model 5 from dDAT (PDB ID 4XPA), model 8 from dDAT (PDB ID 4M48), and model 12 from hSERT (PDB ID 5I6X) for NE docking studies.

**TABLE 1:** SLC6 model analysis.

**Physical properties**					

	**Backbone**		**Side-chain**	**All-atoms**	**Statistical potential**
**MODEL**	**Ramachandran outliers**	**Cβ outliers**	**Rotamer outliers**	**Molprobity score**	**QMEAN4 Z-score**
1 dDAT (4XPA)	9	11	16	3.15	−3.13
2	4	4	19	3.11	−3.72
3	6	4	11	2.86	−3.99
4	5	8	19	3.19	−3.48
5	1	5	14	2.99	−2.64
6 dDAT (4M48)	9	15	27	3.33	−3.05
7	7	7	21	3.15	−3.24
8	7	1	15	3.07	−2.24
9	4	4	10	2.90	−3.41
10	11	8	14	3.14	−3.89
11 hSERT (5I6X)	4	3	14	2.98	−3.75
12	2	3	15	2.96	−2.44
13	6	1	12	2.89	−3.46
14	6	3	12	2.89	−3.64
15	5	6	13	2.90	−3.55

**TABLE 2:** Ramachandran and C_β_ outliers in homology models.

**Model**	**Ramachandran outliers**	**Cβ outliers**
1 dDAT (4XPA)	L25, A27, S182, K189, L190, K204, Y205, T208, N596	Q118, N181, P183, C185, P188, K204, I327, L386, L390, I461, and V569
2	D187, K189, L584, A602	L191, L412, P513, and V569
3	L25, K29, Q43, N184, Y205, L584	N153, P188, L191, and L413
4	R56, D187, L190, D546, and L584	P55, R81, I389, I428, I490, R500, P513, and V569
5	A53	R56, P188, Q393, Q488, and V569
6 dDAT (4M48)	A47, F133, S194, H199, Y202, S203, F207, T208, and H599	I96, I103, W128, F133, N153, P188, Y202, K204, K206, F207,A210, Q393, S401, Q488, and V569
7	T19, R56, D187, K189, V195, I549, and P594	I96, Q113, N153, L191, L238, I389, and V569
8	D52, D187, L190, V195, H199, N596, Q597	M411
9	R56, T58, D187, V195	T58, N192, L238, and P513
10	T19, Q54, R56, K189, V195, H199, S203, K204, F550, P551, and Q608	R56, K189, L191, P209, R341, I389, D434, and P551
11 hSERT (5I6X)	A53, Q54, L190, and V374	W128, F150, and R442
12	Q54, V374	N333, I96, and Q54
13	D50, D52, A53, P188, V374, and G383	W128
14	V42, R49, P188, V374, G383, and H598	N333, L150, and L190
15	V42, A47, P188, V374, and G383	W128, P133, F150, S224,D342, K463

### Mode of NE-hNET Interaction

**FIGURE 2:**
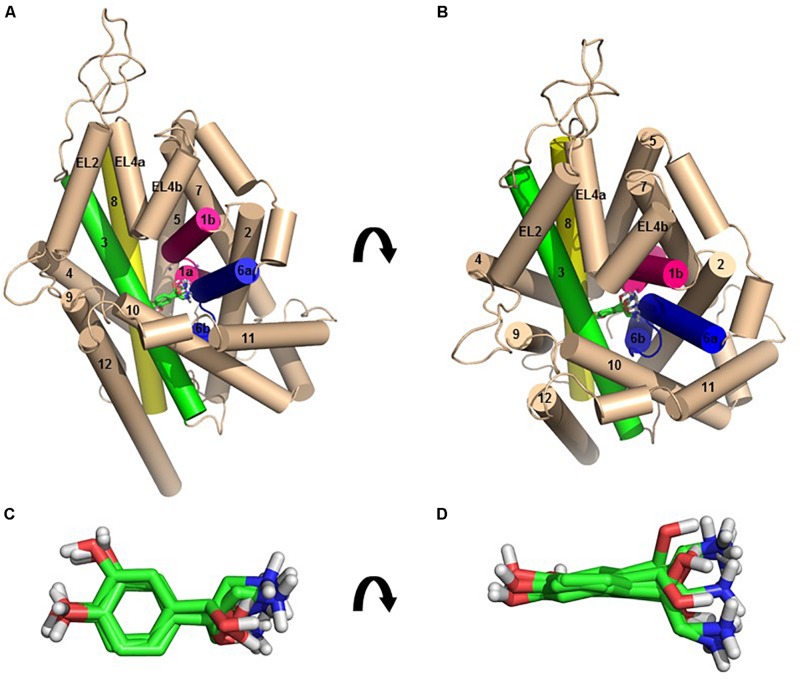
The homology model of NE-hNET complex. Six docking poses for NE in the hNET model based on the dDAT (4M48) structure, viewed from side (**A**, above) or from an extracellular view (**B**, below). TMHs 1, 3, 6, and 8 are shown as pink, green, blue, and yellow cylinders, respectively. The other TMHs and intra- and extracellular loops have been shown as light cream cylinders for clarity. Sodium ions have been represented as purple spheres and NE as green sticks. **(C)** Top view of the six NE docking poses, shown as green sticks. **(D)** Side view of the six NE docking poses, shown as green sticks.

**FIGURE 3:**
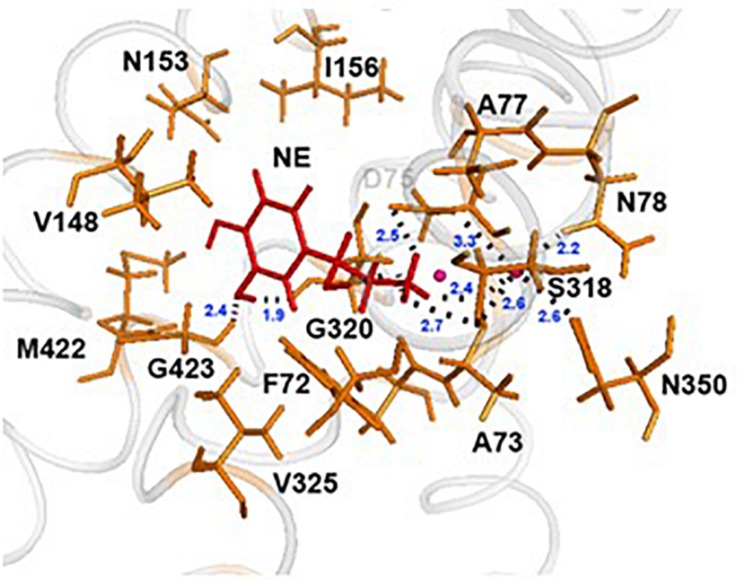
NE docking to the hNET. Intermolecular interactions of NE and hNET residues in NE’s proposed central binding site. NE is represented as sticks and colored red, whereas hNET residues are represented as sticks and colored orange. Na^+^ is shown as pink spheres.

**FIGURE 4:**
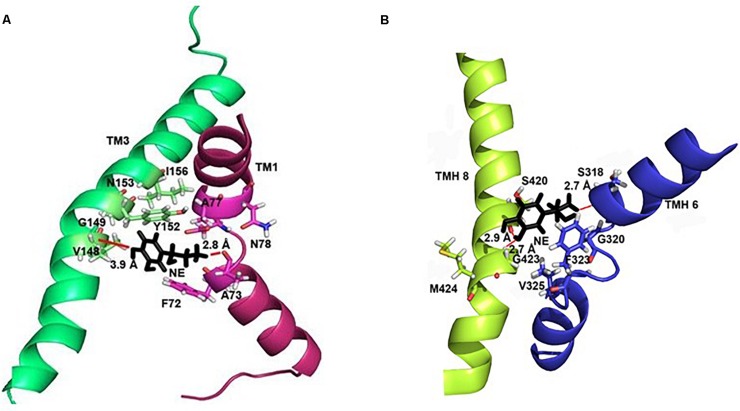
Specific intermolecular interactions between NE and hNET residues. **(A)** Specific intermolecular interactions between NE and residues in TMH1 and TMH3. **(B)** Specific intermolecular interactions between NE and residues in TMH6 and TMH8. NE is shown as black sticks. Residues selected for mutational analysis are highlighted as sticks. Na^+^ ions are not shown for clarity. Hydrogen bonds are shown as red lines part of NE.

**FIGURE 5:**
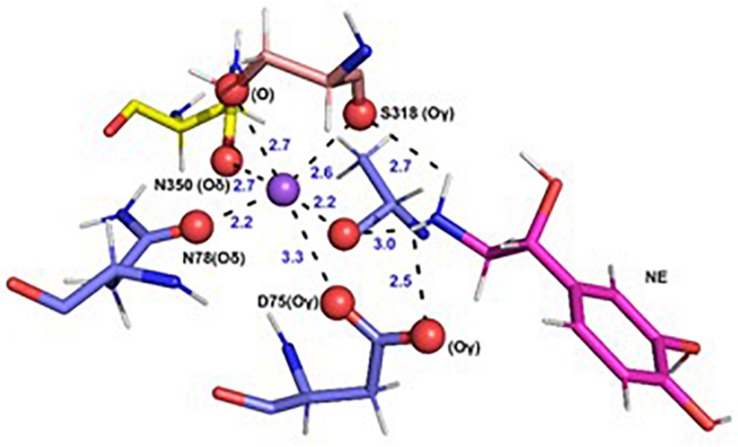
Specific intermolecular interactions of Na^+^ with NE and hNET residues. Octahedral co-ordination of Na^+^ with NE and hNET residues as predicted in the docked hNET homology model. Na^+^ is shown as a purple sphere. NE and hNET residues are represented as sticks. NE is colored pink; residues belonging to TMH1, TMH6, and TMH7 are shown in light blue, light orange, and yellow, respectively. Oxygen, hydrogen, and nitrogen atoms are represented in red, white, and dark blue, respectively.

In each of the three hNET models constructed (Supplementary Figure S1), NE docks centrally in the same orientation and is surrounded by residues from TMHs 1, 3, 6, and 8 (Figure 2). The NE binding cavity from each docking study comprised the same eighteen residues, F72, A73, D75, A77, N78, V148, Y152, N153, I156, S318, G320, F323, V325, F329, N350, S420, G423, and M424 (Figures 3, 4). Of these, A73, A77, G320, S420, G423, and M424 were predicted to make direct hydrogen bond interactions with NE. The amine group of NE forms H-bonds with the main chain oxygen of A73 at 2.8 Å, the main chain amide nitrogen of A77 at 3.0 Å, the main chain oxygen of S318 at 2.7 Å, and the main chain nitrogen of G320 at 3.1 Å (Figures 3, 4). The oxygen atom in the first hydroxyl of the catechol group forms H-bonds with the main chain oxygen of S420 at 2.9 Å and with the main chain nitrogen of G423 at 2.7 Å. F72, A77, V148, Y152, F323, V325, F329, and G423 have hydrophobic interactions with the aryl portion of NE at 3.1 Å, 3.0 Å, 3.3 Å, 3.1 Å, 4.7 Å, 4 Å, 5.7 Å, and 2.4 Å, respectively (Figure 3). Finally, octahedral coordination of NE is provided by Na_1_^+^ through the carbonyl oxygen of A73 (TMH1) and S318 (TMH6), the hydroxyl oxygen of S318 (TMH6) and the side-chain carbonyl oxygen of D75 (TMH1), N78 (TMH1), and N350 (TMH8) (Figure 5).

### Western Blot Results

**FIGURE 6:**
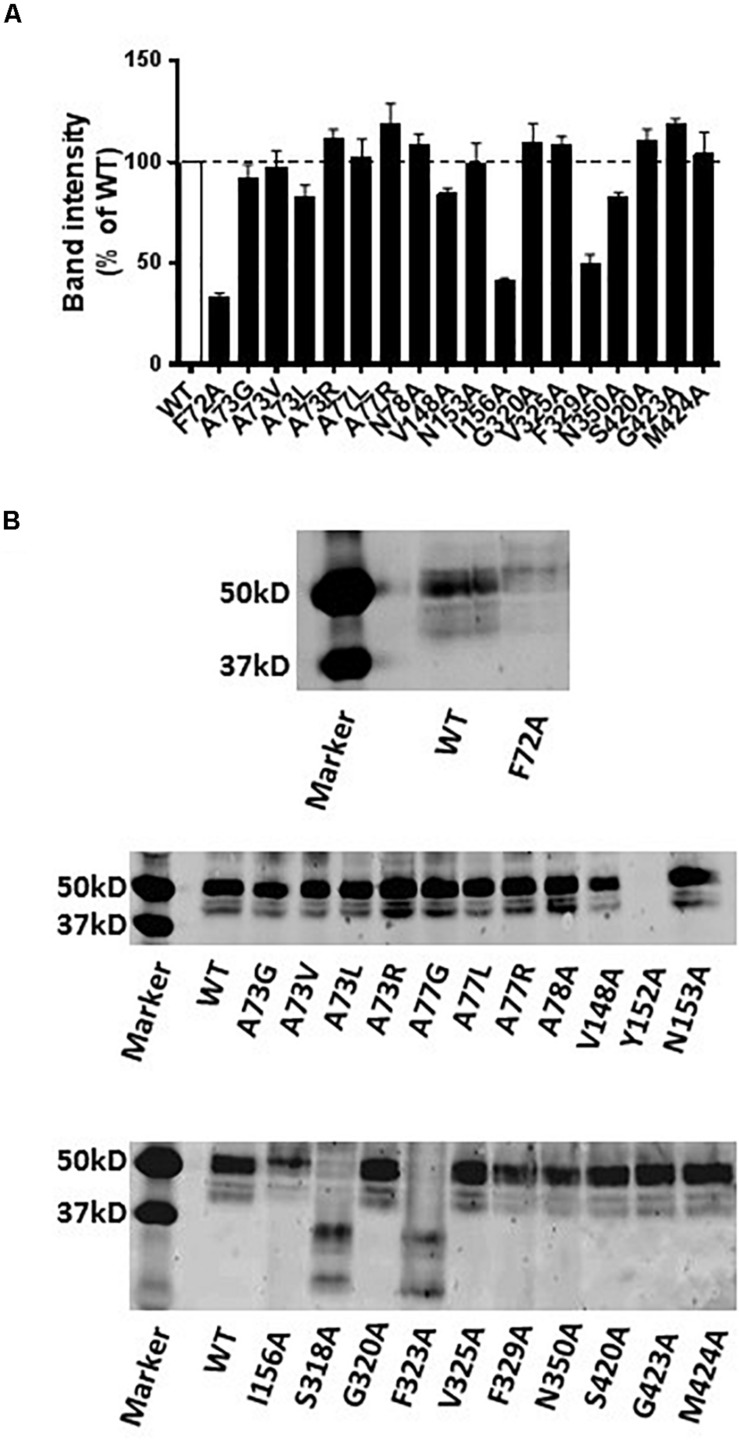
Immunodetection results of WT and mutant hNETs indicating transporter expression levels. **(A)** Bar graph indicating the expression levels of WT and hNET mutants. Values are means ± SEM of 3–4 separate experiments for mutants and WT hNET. **(B)** Representative western blot results.

Western blot analysis revealed that the A73G, A73V, A73L, A73R, A77L, A77R, N78A, V148A, N153A, G320A, V325A, N350A, S420A, G423A, and M424A mutants of hNET had similar expression levels compared to WT hNET when expressed in COS-7 cells. In contrast, the Y152A mutant did not express and the F72A, I156A, S318A, F323A, and F329A mutants showed reduced expression levels compared with WT hNET (Figure 6).

### NX Binding Assay Results

**FIGURE 7:**
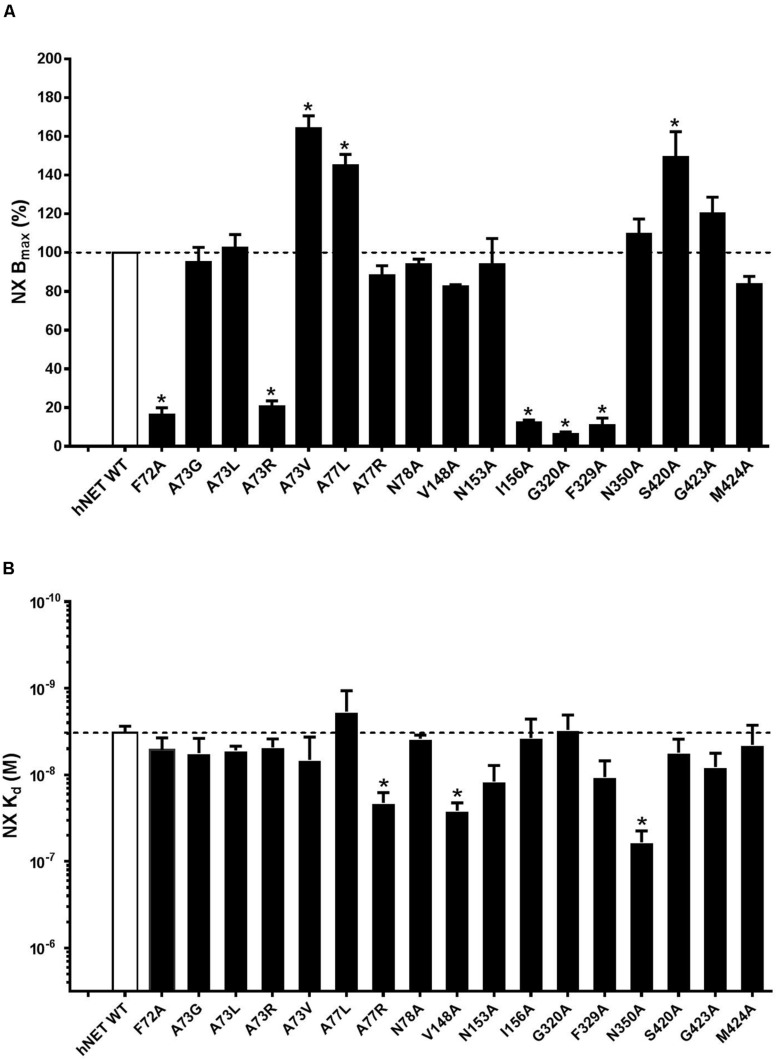
Effect of hNET mutants on NX binding. Comparison of WT and hNET mutants **(A)** B_max_ and **(B)** K_d_ for NX determined from saturation binding curves using increasing concentrations of the radiolabeled hNET antagonist [^3^H]-NX (0.5–50 nM). Non-specific binding was determined in the presence of 200 μM NX. Values are means ± SEM of 3–4 separate experiments for mutants and *n* = 15 for WT, each performed in triplicate. *Significant mutant effects compared with WT hNET, analyzed by Dunnett’s multiple comparisons test after one-way ANOVA.

Nisoxetine binding properties were determined for WT and mutant hNETs transiently expressed in COS-7 cells (Table 3). The B_max_ and K_d_ value for [^3^H]-NX at WT and mutant receptors were determined on cell membranes to establish expression levels and NX affinity, respectively. Specific binding (i.e., B_max_ values) of [^3^H]-NX (1 h, 4°C) significantly decreased for F72A (sixfold), A73R (fivefold), I156A (ninefold), G320A (14-fold), and F329A (12-fold) and increased for A73V (twofold), A77L (twofold), and S420A (twofold) compared to WT (Figure 7A). These changes in the B_max_ values are in general agreement with the band intensity levels observed in the western blot analysis except for the G320A mutant. The binding affinity (K_d_) of NX was reduced at A77R (sixfold), V148A (sevenfold), and N350A (12-fold) compared to WT (K_d_ = 3.67 ± 0.54, *n* = 15) (Figure 7B).

**TABLE 3:** Norepinephrine (NE) uptake and Nisoxetine (NX) binding affinity at WT and mutant hNET.

	**Position**	**[^3^H]-NE K_m_ (μM)**	**[^3^H]-NX K_d_ (nM)**	**[^3^H]-NE V_max_ (%)**	**[^3^H]-NX B_max_ (%)**	**(V_max_/B_max_) (%)**
WT		1.580.33	3.670.54	100	100	100
F72A	TMH1a	2.501.29	6.353.15	14.835.36*	16.493.48*	90.08.84
A73G	Loop1a-1b	7.741.59*	7.584.38	41.203.66*	95.337.44	43.011.10*
A73L	Loop1a-1b	0.631.97	5.280.59	4.670.69*	102.606.80	4.607.49*
A73R	Loop1a-1b	1.090.29	5.311.99	0.650.47*	21.174.93*	3.105.40*
A73V	Loop1a-1b	4.800.58*	4.391.09	58.304.06*	164.216.43*	35.510.49*
A77L	TMH1b	5.101.10*	2.380.91	1.020.58*	145.305.47*	0.706.05*
A77R	TMH1b	1.500.30	22.475.62*	7.302.47*	88.275.00	8.007.47*
N78A	TMH1b	0.930.69	7.173.92	17.051.05*	96.501.50	18.02.55*
V148A	TMH3	2.741.28	26.338.78*	28.795.95*	82.800.61	35.06.56*
N153A	TMH3	3.650.09*	14.215.43	3.621.82*	94.0013.34	3.8515.16*
I156A	TMH3	1.920.77	5.753.87	5.050.83*	11.240.76*	45.01.59*
G320A	TMH6	3.120.13*	N.D.	8.700.50*	6.681.83*	130.22.33*
V325A	TMH6	1.380.44	N.D.	88.735.76	N.D.	N.D.
F329A	TMH6	0.900.39	16.504.50	4.600.65*	8.595.24*	53.05.89*
N350A	TMH8	0.360.22*	45.165.22*	3.301.40*	112.5012.50	2.9013.90*
S420A	TMH8	0.450.04*	7.922.06	65.8711.45*	149.2522.45*	44.133.90*
G423A	TMH8	5.960.89*	11.931.41	78.531.35	120.408.24	65.29.59*
M424A	TMH8	0.830.09*	7.054.85	68.507.65	83.704.09	81.811.74*

N.D., not determined. Values are the mean ± SEM, of 3–4 independent experiments for hNET mutants and *n* = 15 for WT, each performed in triplicate. *Significant mutant effects compared with WT hNET.

### NE Transport and Affinity

**FIGURE 8:**
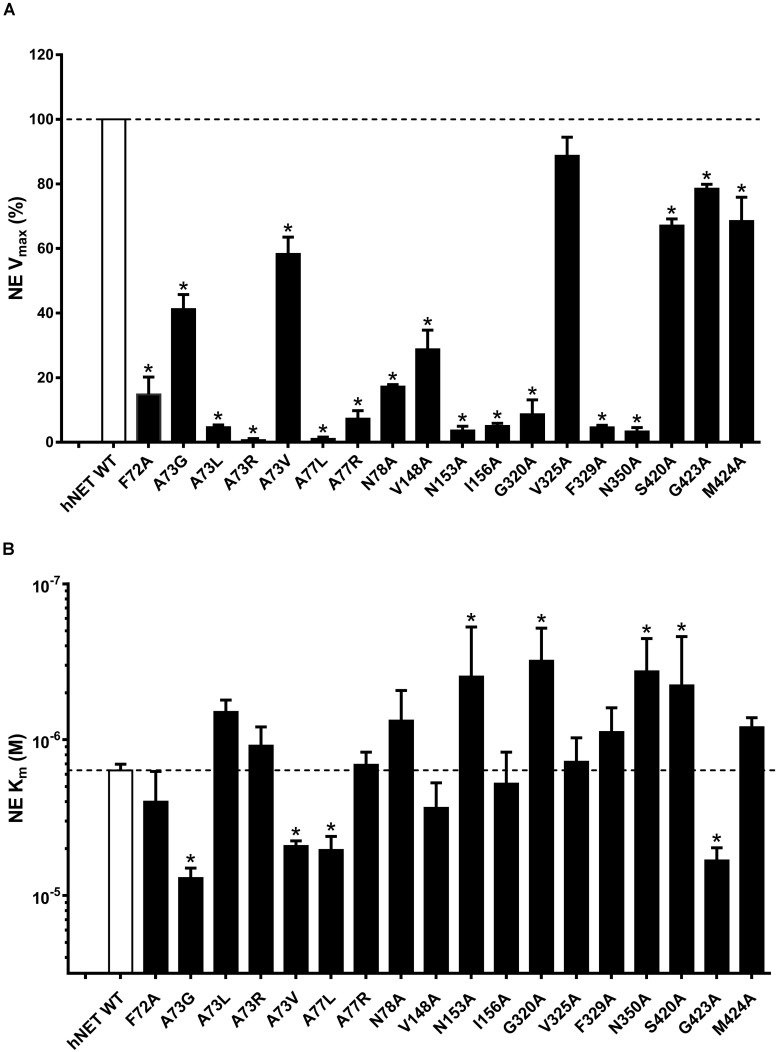
Effect of hNET mutants on NE uptake. Comparison of WT and hNET mutants **(A)** V_max_ and **(B)** K_m_ for NE determined from saturation uptake curves, where non-specific uptake was determined in the presence of 200 μM NX. Values are means ± SEM of 3–4 separate experiments for mutants and *n* = 15 for WT, each performed in triplicate. *Significant mutant effects compared with WT hNET, analyzed by Dunnett’s multiple comparisons test after one-way ANOVA.

Specific uptake of [^3^H]-NE (10 min, 37°C) was saturable in COS-7 cells expressing hNET WT and all hNET mutants (Table 3). Significant reduction in V_max_ values were observed for F72A (sevenfold), A73G (twofold), A73L (21-fold), A73R (154-fold), A73V (twofold), A77L (98-fold), A77R (14-fold), N78A (sixfold), V148A (threefold), N153A (28-fold), I156A (20-fold), G320A (11-fold), F329A (22-fold), N350A (30-fold), and S420A (twofold) compared with hNET WT (Figure 8A). The K_m_ values increased for A73G (fivefold), A73V (threefold), A77L (threefold), N153A (twofold), G320A (twofold), G423A (fourfold), decreased for N350A (fourfold), and S420A (fourfold) compared to the WT (Figure 8B). All other mutants showed no significant change compared to WT V_max_ (100%) or K_m_ (1.58 ± 0.33 nM; *n* = 18).

## Discussion

Substrate binding to the S1 is considered a pivotal step in transporter function. This site lies deep within the transporter and overlaps with the S1 of orthosteric inhibitors (Schlessinger et al., 2011; Sørensen et al., 2012). The S1 pocket is lined by two unwound helical loops positioned centrally in TMH1 and TMH6 that provide structural flexibility required for the transport of substrate (Yamashita et al., 2005). Our model revealed that the S1 binding pocket in hNET is relatively small, restricting the size of the ligands that can interact at this site. Importantly, the unwound helical segments in S1 seen in the hNET model and the NSS crystal structures, allow residues to adopt an extended conformation with their main chain carbonyl oxygen and nitrogen atoms exposed for interactions with substrate and inhibitors. In this study, we docked NE in hNET models 5, 8, and 12 to identify 15 likely functional determinants of S1 associated with TMH1, 3, 6, and 8, that were systematically mutated to alanine to determined their functional role.

Transmembrane helice 1 is a highly conserved “signature” region of the SLC6 transporters. Our docking model revealed that four residues (F72, A73, A77, and N78) in TMH1 of hNET contributed to the S1 pocket. Whereas F72 formed hydrophobic interactions with the aryl moiety of NE at 3.1 Å, the F72A mutant produced an equal drop in both the transport function and protein expression levels, indicating this residue contributed little to overall transport function but may play a structural role in NET. In support, F72V-NET was non-functional (Liu et al., 1998) and F72Y-NET again reduced both transport and expression (Andersen et al., 2011). Other studies suggesting F72 had a direct functional role in transport (Liu et al., 1998; Andersen et al., 2011; Schlessinger et al., 2011; Koldsø et al., 2012) failed to consider the influence of expression levels on transport. F72 also contributes to the cocaine and antidepressant binding site (Lin et al., 1999), with the recent nortriptyline-dDAT co-crystal structure revealing that the main chain carbonyl of F43 in dDAT (corresponding to F72 in hNET) forms a hydrogen bond with the amino group of nortriptyline at 2.7 Å. Y95 in SERT (corresponding to F72 in hNET) also contributes to interactions with antidepressants (Henry et al., 2006). However, the F72A mutant did not affect NX affinity as seen from NX binding studies (<2- fold change), suggesting it is not a part of the NX binding site.

A73 is positioned at the beginning of the unwound loop region of TMH1a, where its main chain oxygen is observed to form a hydrogen bond with the amine of NE (at 2.8 Å) and the Na_1_^+^ ion (at 2.2 Å). The A73G- and A73V-hNET mutants had reduced (5- and 3-fold, respectively) K_m_ for NE and also significantly reduced transport, without affecting expression levels. Replacing alanine with the larger leucine and arginine (A73L- and A73R-hNET) had little effect on NE K_m_ but almost completely abolished transport rate, suggesting these mutants do not contribute directly to NE binding but disrupt conformational changes required for transport. The A73G, A73V and A73L mutants had similar B_max_ and K_d_ values for NX as WT hNET, whereas the A73R mutant had a reduced B_max_, likely linked to protein destabilization, that also influenced protein expression as showed by Western blot. The crystal structure of LeuT with leucine bound revealed that A22 in LeuT (corresponding to A73 in hNET) was involved in the octahedral co-ordination of Na^+^ via its carbonyl oxygen, supporting a structural role as well as providing hydrogen bonding to the amino group of leucine via its main chain carbonyl oxygen (Yamashita et al., 2005).

The A77 residue in TMH1 is positioned at the end of the unwound loop before TMH1b, where its side chain points into the binding pocket toward TMH6. Our model predicts A77 to form a hydrophobic interaction with NE via its benzyl side chain (at 3.0 Å) and a hydrogen bond via the amide nitrogen of its main chain (at 3.0 Å). The A77G mutant has been tested earlier and shown to halve the transport rate (Andersen et al., 2011). Both the A77 mutants of hNET we tested (A77L and A77R) showed negligible transport. A77L had a threefold loss in NE potency (i.e., greater K_m_ value), whereas A77R showed no change in NE potency (i.e., no change in K_m_ value) compared to WT. B_max_ values with respect to WT were increased for A77L and similar for A77R. A77R showed a sixfold loss in the binding affinity of NX. These results suggest that like A73, A77R influence conformational changes relating to transport, perhaps through its role in maintaining the unwound loop connecting TMH1a-TMH1b. The crystal structure of LeuT with substrate leucine bound showed that G26 (corresponding to A77 in hNET) directly interacted through its amide nitrogen with the carboxyl group of leucine (Yamashita et al., 2005), similar to the predicted interactions for A77 in our hNET model. A77 in hNET and its corresponding residues A81 in hDAT and G100 in hSERT have previously been tested for their involvement in defining selective serotonin reuptake inhibitor (SSRI) specificity as they are the only non-conserved residues in the halogen binding pocket of SSRIs (Zhou et al., 2009). This study showed that the different SSRI halogens sertraline, R-fluoxetine and S-fluoxetine bind to the halogen binding pocket that is conserved across LeuT, hNET, hDAT, and hSERT except for this position (Zhou et al., 2009). Mutating A77 in hNET or A81 in hDAT to a glycine (A77G and A81G, respectively) increased their affinities to all the three SSRIs, indicating a direct involvement of this position in determining the protein’s antidepressant specificity (Zhou et al., 2009). Thus, residue A77 seems to be an important determinant in both substrate transport and SSRI binding.

Our docking model of NE in hNET also showed that N78 contributed to the NE binding pocket by octahedral coordination of Na_1_^+^ through its side-chain carbonyl oxygen. In agreement with our docking model, the alanine mutant of N78 (N78A) almost completely abolished NE transport without affecting the binding affinity of NE or NX. This result suggests that octahedral coordination of Na_1_^+^ might be critical for substrate transport. In addition, N78 could potentially be interacting with V244 in TMH7, a residue also important for efficient transport. We hypothesize that their interaction could play a role in transporter destabilization. Since N78 is conserved across the transporter SLC6 superfamily except in LeuT, which has a valine, the residue at this position is likely to play an important role in structure and function of the transporter.

Our model also shows that V148, Y152, N153, and I156 in TMH3 contribute a hydrophobic surface to the NE binding pocket. A possible hydrophobic interaction of V148 with the aryl group of NE (3.3 Å) was confirmed experimentally as the alanine mutant V148A reduced transport rate ∼threefold and NE binding affinity ∼3.5-fold. The V148A mutant also reduced NX binding affinity sevenfold, indicating that V148 might form part of the NX binding pocket. V148 in hNET and its corresponding isoleucine residue in SERT are major determinants of SERT/NET selectivity for selective norepinephrine reuptake inhibitors (SNRIs) and serotonin reuptake inhibitors (SSRIs) (Henry et al., 2006; Sørensen et al., 2012). The corresponding residue V152 in hDAT has been found to be involved in DA and cocaine binding (Beuming et al., 2008). Thus, V148 is involved in the binding of substrates and orthosteric ligands binding.

Hydrogen bonding between Y152 and D75 in hNET also appears to be an important factor for maintaining a functional transporter and for NE binding. The Y152A hNET mutant abolished protein expression, whereas the previously studied Y152F mutant had only 6% transporter activity (Andersen et al., 2011). The corresponding D79 and Y156 in DAT are also essential for DA binding since mutating either the D79 or Y156 in DAT significantly affected DAT binding and transporter function (Beuming et al., 2008).

N153 also appeared critical to NE binding and transport since the N153A mutant had reduced transport but increased NE affinity. The presence of this isolated polar residue in the S1 hydrophobic pocket suggests it may provide the appropriate level of affinity for NE for efficient transport. B_max_ and K_d_ values for NX were unaffected by this mutant, suggesting that transporter structure was unaffected. The N153S mutation has been shown to alter the hydrogen bonding properties and to reduce the transport activity to 19% of WT hNET and to decrease the binding affinity of NE, with no effect on norepinephrine reuptake inhibitor (NRI) binding (Sørensen et al., 2012). However, the corresponding N157 in DAT does not seem to be involved in DA binding but instead contributes to cocaine binding (Beuming et al., 2008). In contrast, the corresponding N177 in SERT is involved in 5-HT binding, SSRI and SNRI binding (Sørensen et al., 2012). Given asparagine is involved in NE and 5-HT but not DA binding might explain why SNRIs do not inhibit hDAT.

Residues S318, G320, F323, and V325 in TMH6 were also identified as part of the NE S1 binding site. Our model predicted S318 carbonyl and hydroxyl oxygen were involved in hydrogen bonding with Na_1_^+^ and potentially with NE. In support of this, the corresponding residue T254 in LeuT also had hydrogen bond interactions with leucine and Na_1_^+^ (Yamashita et al., 2005). It was therefore expected that the S318A mutation would directly affect NE and Na_1_^+^ binding and thus disrupt normal transport function. However, western blot analysis revealed a decreased expression of the S318A and F323A mutants, possibly related to incorrect folding, suggesting that the S318 and F323 residues would play key structural roles contributing also to transporter function.

Residue G320 is positioned close to docked NE and was thus predicted to directly affect NE binding. Consistent with this view, a conservative change to alanine at this position almost completely abolished transport, while increasing NE binding affinity, suggesting NE might get trapped in the S1 binding site. Alternatively, since G320 is positioned in the unwound loop of TMH6, which plays a significant role in the conformational changes during the transport cycle, the mutation to alanine might stabilize the transporter in one particular conformation to reduce transport. Such an effect might slow ligand binding and explain the reduced ^3^H-NX B_max_ value despite protein expression levels being at WT levels. In support, mutating the corresponding G338 in SERT to cysteine produced similar effects shown to arise from stabilization of an outward-open conformation that prevented transporter movement (Field et al., 2010).

Our model shows V325 in THM6 positioned at the outer edge of the S1 binding site and remote from docked NE. Consistent with its predicted minimal role, mutating it to alanine had little impact on the NE transport and affinity, supporting the docking observation of NE.

The remaining three residues identified to form part of the S1 binding site are S420, G423, and M424 in TMH8. S420 and M424 form a hydrophilic pocket that accommodates the two hydroxyls of NE. Docking results indicated the meta-hydroxyl of NE would form a hydrogen bond with the side chain of S420 and G423, whereas the para-hydroxyl would form hydrogen bonds with M424. The S420A and M424A mutants had increased affinity for NE along with reduced NE transport rate, indicating that the hydrophilicity at these positions is essential in maintaining the normal transport rate of the transporter. The 5-hydroxyl group of 5-HT and the meta-hydroxyl group of DA have also been shown to interact with the hydrophilic pocket around S438 (hSERT) and S422 (hDAT), respectively (Koldsø et al., 2012). The G423A has reduced binding affinity for NE and reduced transport rates, confirming the docking result and the hypothesis.

Finally, N350 in TMH8 is predicted from our docking model to participate in the octahedral coordination of Na_1_^+^ via its side-chain carbonyl oxygen and is also anticipated to indirectly affect transporter function. Similar to what was observed for the G320A mutant, the N350A mutant abolished NE transport while increasing NE affinity, despite B_max_ values for NX binding remaining at WT levels. However, affinity for NX was reduced 12-fold. Thus, we predict that the N350A mutant might stabilize conformations favoring substrate binding over antidepressant binding that prevents transporter cycling. N350 is conserved across the transporter SLC6 superfamily, with the corresponding N368 in SERT being highly sensitive to non-conservative replacements (Penado et al., 1998). The hydrogen bond between N350 and the Na^+^ ion appears critical to transport function, since the N350L and N350I mutations also significantly reduced transport, whereas the N350D mutation maintained near WT transporter function (Penado et al., 1998).

## Conclusion

In conclusion, this study on hNET provides new insights into the structural features contributing to substrate specificity. Our docking studies showed that the catechol hydroxyl of NE did not form intermolecular interactions with the hNET, while the catechol benzene, catecholamine and β-hydroxyl of NE formed direct interactions with hNET. This suggests that the catechol hydroxyl part of NE is not essential for transport, explaining why hNET can transport both NE and DA. In contrast, hDAT does not transport NE, suggesting that the size of the catechol tail is key determinant of hDAT monoamine substrate specificity. We found that the residues involved in Na_1_^+^ binding are critical for NET function with A73, N78, and N350 affecting the NE binding affinity as well. We identified that 8 of 15 residues lining the primary binding site of NE in hNET decreased (A73, A77, N153, G320, and G423) or increased (N350, S420, and M424) NE affinity. 11 of 15 residues reduced NE transport, including several residues (A73, A77, N78, V148, Y152, and F323) that are conserved across monoamine transporters. Several mutations appear to restrict transporter cycling in a substrate (N350A, S420A, and M424A) preferring conformation, which may help in identifying the structure of additional transporter conformational states and more specific transporter inhibitors.

## Data Availability Statement

The raw data supporting the conclusions of this article will be made available by the authors, without undue reservation, to any qualified researcher.

## Author Contributions

PJ and RL participated in research design. PJ conducted experiments. PJ and LR performed the data analysis. PJ, LR, and RL wrote or contributed to the writing of the manuscript.

## Conflict of Interest

The authors declare that the research was conducted in the absence of any commercial or financial relationships that could be construed as a potential conflict of interest.

## Abbreviations

5-HT: serotonin
A: L-Alanine
ADHD: attention deficit hyperactivity disorder
CNS: central nervous system
DA: dopamine
DAT: dopamine transporter
GABA: γ-aminobutyric acid
hNET: human norepinephrine transporter
LeuT: leucine transporter
NE: norepinephrine
OCD: obsessive compulsive disorder
S1: high affinity binding site
SERT: serotonin transporter
SLC: solute carrier
TMHs: transmembrane helices
WT: wild type.
