# Supplementary material for: Structure-Function of the High Affinity Substrate Binding Site (S1) of Human Norepinephrine Transporter
Source: Front Pharmacol. 2020 Mar 5;11:217. doi: 10.3389/fphar.2020.00217 (PMC7066499; doi:10.3389/fphar.2020.00217)

Supplementary Figure 1. Alignment of hNET homology models based on dDAT (PDB ID 4M48) in blue, dDAT (PDB ID 4XPA) in green and hSERT (PDB ID 5I6X) in yellow.

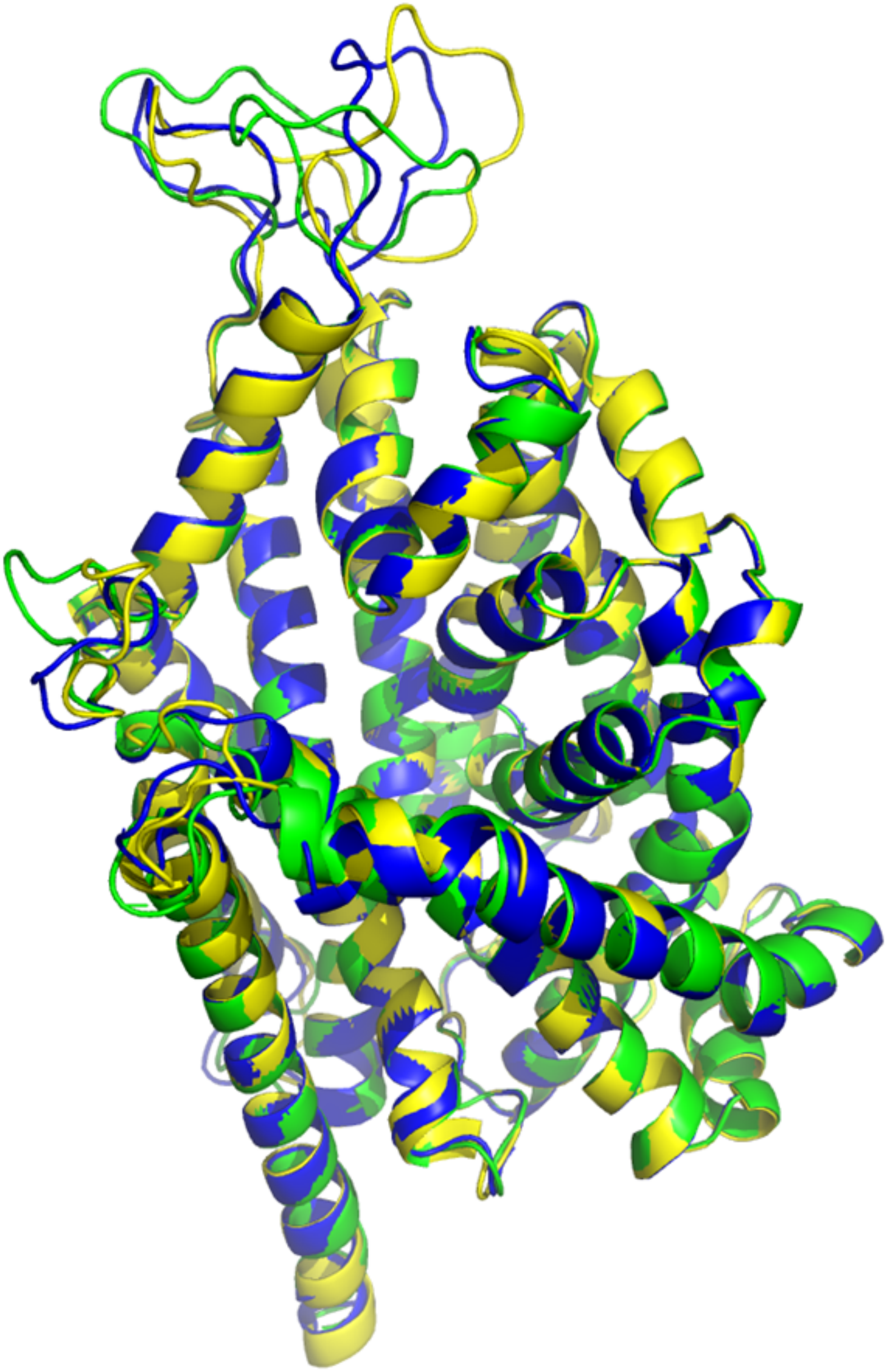

Supplement: Supplementary file 1 [file Image_1.pdf]
