# Supplementary material for: Structure-Function of the High Affinity Substrate Binding Site (S1) of Human Norepinephrine Transporter
Source: Front Pharmacol. 2020 Mar 5;11:217. doi: 10.3389/fphar.2020.00217 (PMC7066499; doi:10.3389/fphar.2020.00217)
Supplement: Supplementary file 2 [file Table_1.pdf]

Supplementary Table 1. hNET SNP effects on pharmacology and pathology.

Mutation	Pharmacological effect	Pathology	Reference
V69I	No effect in K_m and V_{max} for NE transport; no change in desipramine potency		(Runkel, Brüss et al. 2000)
T99I	No effect in K_m and V_{max} for NE transport; no change in desipramine potency		(Runkel, Brüss et al. 2000)
R121Q	Greater impact on NE transport than DA transport	Long QT syndrome (disorder of the heart's electrical activity)	(Hahn, Mazei-Robison et al. 2005, Hahn and Blakely 2007)
V244I	Little effect on hNET expression levels or transport of NE and DA.		(Hahn, Mazei-Robison et al. 2005)
V245I	No effect in K_m and V_{max} for NE transport; no change in desipramine potency	Attention deficit hyperactivity disorder (ADHD)	(Runkel, Brüss et al. 2000)
N292T	Some loss of NE and DA transport and expression of functional transporter		(Hahn, Mazei-Robison et al. 2005)
V356L	Little effect on hNET expression levels and transport of NE and DA.		(Hahn, Mazei-Robison et al. 2005)
V356S	Increase in DA uptake but decrease in affinity for nortriptyline and desipramine.		(Hahn, Mazei-Robison et al. 2005)
A369P	Complete loss of transport of NE and DA; retained intracellularly.		(Hahn, Mazei-Robison et al. 2005)
N375S	Little effect on hNET expression levels and transport of NE and DA.		(Hahn, Mazei-Robison et al. 2005)
N375P	2-fold increase in NE and DA uptake.		(Hahn, Mazei-Robison et al. 2005)
V449I	No effect in K_m and V_{max} for NE transport; no change in desipramine potency		(Runkel, Brüss et al. 2000)
A457P	5-fold higher affinity for cocaine; two-fold lower affinity for nisoxetine, and unchanged affinity for desipramine. 40% expression of the wild-type hNET; 50-fold higher K_m for NE		(Hahn, Robertson et al. 2003)
K463R	Little effect on hNET expression levels and transport of NE and DA		(Hahn, Mazei-Robison et al. 2005)
G478S	4-fold increase in K_m for NE uptake but no change in V_{max}; no change in desipramine potency		(Runkel, Brüss et al. 2000)
F528C	Increase in ratio of NE to DA transport and V_{max} to K_m for NE transport; no change in K_i values for NE competition of NX binding or in DA uptake; increase in translocation of NE	Major depression	(Hahn, Mazei-Robison et al. 2005, Haenisch, Linsel et al. 2009)
Y548H	20% loss of NE and DA transport and expression of functional transporter.		(Hahn, Mazei-Robison et al. 2005)
I549T	Decrease in protein expression but no change in NE and DA transport.		(Hahn, Mazei-Robison et al. 2005)

Haenisch, B., K. Linsel, M. Bruss, R. Gilsbach, P. Propping, M. M. Nothen, M. Rietschel, R. Fimmers, W. Maier, A. Zobel, S. Hofels, V. Guttenthaler, M. Gothert and H. Bonisch (2009). Association of major depression with rare functional variants in norepinephrine transporter and serotonin$_{1A}$ receptor genes. *American Journal of Med Genet B* **150B**: 1013-1016.

Hahn, M. K. and R. D. Blakely (2007). The functional impact of SLC6 transporter genetic variation. *Annual Review of Pharmacology and Toxicology* **47**: 401-441.

Hahn, M. K., M. S. Mazei-Robison and R. D. Blaokely (2005). Single nucleotide polymorphisms in the human norepinephrine transporter gene affect expression, trafficking, antidepressant interaction, and protein kinase C regulation. *Molecular Pharmacology* **68**(2): 457-466.

Hahn, M. K., M. S. Mazei-Robison and R. D. Blakely (2005). Single nucleotide polymorphisms in the human norepinephrine transporter gene affect expression, trafficking, antidepressant interaction, and protein kinase C regulation. *Molecular Pharmacology* **68**(2): 457-466.

Hahn, M. K., D. Robertson and R. D. Blakely (2003). A mutation in the human norepinephrine transporter gene (SLC6A2) associated with orthostatic intolerance disrupts surface expression of mutant and wild-type transporters. *The Journal of Neuroscience* **23**(11): 4470-4478.

Runkel, F., M. Brüss, M. M. Nöthen, G. Stöber, P. Propping and H. Bönisch (2000). Pharmacological properties of naturally occurring variants of the human norepinephrine transporter. *Pharmacogenetics and Genomics* **10**(5): 397-405.
