# Supplementary material for: Structure-Function of the High Affinity Substrate Binding Site (S1) of Human Norepinephrine Transporter
Source: Front Pharmacol. 2020 Mar 5;11:217. doi: 10.3389/fphar.2020.00217 (PMC7066499; doi:10.3389/fphar.2020.00217)
Supplement: Supplementary file 3 [file Table_2.pdf]

Supplementary Table 2. Functionally conserved residues across the SLC 6 family.

**hNET**	**DAT**	**SERT**	**LeuT**
A73	A77	A96	A22
A77	A81	G100	G26
N78	N82	N101	N27
V148	V152	I172	V104
N153	N157	N177	N109
I156	I160	M180	I112
G320	G323	G338	S256
F329	Y332	F347	I265
N350	N353	N368	A286
S420	S423	T427	L356
G423	G426	G430	I359
M424	S427	L431	-----
